# Evaluation of coffee husks in pellet bedding, performance characteristics, *footpad dermatitis* scoring, and meat quality of broiler ducks

**DOI:** 10.1007/s11259-023-10196-w

**Published:** 2023-08-21

**Authors:** Jakub Biesek, Sebastian Wlaźlak, Mirosław Banaszak, Małgorzata Grabowicz

**Affiliations:** Department of Animal Breeding and Nutrition, Faculty of Animal Breeding and Biology, Bydgoszcz University of Science and Technology, Mazowiecka 28, 85-084 Bydgoszcz, Poland

**Keywords:** Waterfowl, Litter material, Coffee waste, Performance characteristics, Foot pad condition

## Abstract

The study aimed to analyze the chemical composition of pellet bedding made of straw or coffee husks (10, 25, 50%) and the performance characteristics of broiler ducks and *footpad dermatitis*. During rearing, the properties of the bedding and utility features of ducks were analyzed, and the frequency of *footpad dermatitis* (FPD) in ducks was verified. There was a decrease in dry matter from the 28th day of rearing. The crude fiber, NDF, ADF, and nitrogen content decreased compared to fresh bedding, while phosphorus and potassium increased. The highest pH was found in the CH25 and CH50 groups in fresh bedding on day 42 and in CH50 on day 14. High adj. R^2^ was found due to rearing time and bedding material (0.817–0.985). The ducks’ growth rate in the CH25 group was higher at week 6 than in the other groups. In CH10 and CH25 groups, higher carcass weight was found than in group C. In group CH10, a higher weight of pectoral muscles and lower wing proportion was found than in C. In CH25, a higher remains weight was shown than in C and CH50. In CH50, lower water-holding capacity in the pectoral muscles was found than in the other groups. Considering the bedding (the content of nitrogen, phosphorus, and potassium), carcass features, and meat quality (water-holding capacity, intramuscular fat, and water content), it is possible to use 10, 25 or 50% of coffee husks in straw pellets in the rearing of broiler ducks. Due to the FPD, the moisture should be lowered.

## Introduction

Poultry production requires constant monitoring of environmental conditions in livestock buildings (Loshkarev et al. 2019). It is also necessary to use high-quality bedding, which affects the production results, meat quality, and birds’ health (Bodo and Galik 2018; Diarra et al. 2021). The dynamic development of poultry production, mainly the increased number of birds kept on bedding, requires more good raw materials free from contamination. After the production cycle, such bedding could be used as a fertilizer in plant production. However, due to many factors (composition of the feed, type of bedding, density of birds per 1 m^2^ of the area), the bedding components content highly varies (Rogeri et al. 2016).

The properties of bedding are influenced mainly by its chemical composition and the content of individual components (Diarra et al. 2021). A good bedding material should be characterized by, among others: high water absorption, anti-caking properties, low price, easy availability (Garcia et al. 2012), softness, and thermal insulation (Souza et al. 2012). Keeping ducks in bedding is primarily one of the elements of their welfare. Birds can exhibit natural behavior, and high bedding quality (mainly absorption capacity) contributes to the formation of ammonia (Mohammed et al. 2019) and inflammation of the foot pads (FPD) (De Jong et al. 2014; Sonnabend et al. 2022). In addition, the high water absorption capacity of the bedding limits its excessive moisture, which is a favorable environment for the development of many microorganisms (Diarra et al. 2021). Keeping birds in the bedding also promotes better hygiene and reduces stress reactions, which has been confirmed in the example of broiler ducks (Mohammed et al. 2019).

Straw is the most popular bedding material in European poultry production, unlike in the United States, where sawdust is most used (Shepherd et al. 2017). However, new, alternative materials are increasingly being sought for such applications (Diarra et al. 2021). Oketch et al. (2023) researched using coconut peat, sawdust, and rice husks as bedding in the Pekin ducks production. As the authors indicated, rice husks can be a good bedding material with beneficial properties. Scientists have indicated that the region where raw material is produced is essential. For rice-producing countries such as South Korea, where the use of rice husks as bedding material for broiler chickens and ducks is approx. 85%. It also is crucial for materials competitiveness. However, bedding for ducks should be free of contaminants (toxins, molds, pathogens). A threat is the consumption of litter by birds (Farghly et al. 2021; Oketch et al. 2023).

Coffee husks are post-production waste during dry roasting (Cangussu et al. 2021), which can be used in various industrial sectors (Oliveira and Franca 2015). The possibility of their use in producing biofuels, mushrooms, and adsorbents and in feeding farm animals (chicken, cattle, sheep, pigs, and horses) has been verified (Oliveira and Franca 2015). Coffee husks were also tested at the request of the European Commission by EFSA for use to produce new food safe for humans (Turck et al. 2022). Available scientific studies also show the use of coffee husks as bedding for horses (Benezoli et al. 2019), pigs (Caldara et al. 2012), and poultry (Marín et al. 2015; Souza et al. 2018). Since coffee processing is increasing in Poland, managing coffee production waste is part of maintaining a sustainable agri-food market (Głowacka et al. 2018).

Coffee husks contain the most carbohydrates (58–85 g/100 g dry matter). In addition, the presence of proteins, fats, and minerals is also found (Oliveira and Franca 2015). The total fiber content of coffee husks was 65.83 g/100 g (Cangussu et al. 2021). The chemical composition of wheat straw includes, among others: dry matter (89–94%), protein (3.6%), acidic detergent fiber (54%), cellulose (40%), hemicellulose (26%), lignin ( 22.9%), ash (up to 9.9%), calcium (0.18%) and phosphorus (0.05%) (Khan and Mubeen 2012).

The available literature considering the effects of various bedding materials, particularly coffee husks, on the chemical composition of bedding, welfare, and performance of ducks under production conditions in Europe is limited. However, as one of the alternatives (e.g., the commonly used straw) coming from so far little-discussed industry sectors, they could support duck meat producers, mainly through an impact on the welfare and quality characteristics of the product.

The study aimed to analyze the chemical composition of bedding in the form of pellets with a different share of coffee husks and to evaluate broiler ducks’ growth performance, carcass characteristics, meat quality, and foot pad condition.

## Materials and methods

The experiment was carried out following the applicable regulations on the use of animals in science. The study and methods were carried out after obtaining the opinion of the Departmental Animal Welfare Team and the permission of the Experimental Unit of the Bydgoszcz University of Science and Technology (No. 2/2022).

### Animals and experimental design

In the 42 days of experimental rearing, 200 1-day-old male Cherry Valley ducks were used. The average initial body weight was 56.07 ± 1.47. All birds were divided into 4 equal groups (5 replications within 10 birds each). The stocking density did not exceed 17 kg per 1 m^2^. On the first day, an air temperature of 26 °C was used, and then it was gradually reduced to 20 °C. An additional heat source was installed in each of the pens and used until the 4th week of rearing (from 30 °C decreasing). Humidity was 60–65%. Feed and water were used *ad libitum*. Environmental conditions were done according to Biesek et al. (2022a). In all groups, ducks were fed with a commercial compound feed, the composition of which corresponded to the nutritional requirements for this poultry species. The two feeding phases were applied; the first feeding period lasted from day 1 to day 28 when the starter feed was used. From day 29 to day 42, the ducks were fed grower feed.

In the control group (C), wheat straw pellets were used as bedding material. Then, in the experimental groups, pellets with a different share of coffee husks were used, which were 10% (CH10), 25% (CH25), and 50% (CH50), respectively. In all groups, 7 kg of bedding per 1 m^2^ floor area was applied the day before the experiment. The pellet was prepared using the RTH-150 granulator (Peletto, Poznań, Poland) using wheat straw (previously crushed) or wheat straw in combination with the appropriate amount of coffee husks, depending on their percentage in the bedding. Coffee husks were obtained after dry roasting coffee in a roasting plant in the Kuyavian-Pomeranian Voivodeship in Poland. The pellet preparation was carried out under patent application pending procedure number P.44,383. On average, the pellet was characterized by 6 mm in diameter and 2 cm in length.

On the 42nd day of rearing, 10 birds from each group were selected for slaughter, with a body weight similar to the average body weight of birds in a given group (2 replicates each), and slaughtered. The body weight of chosen birds was compared to the average body weight of each replication (pen). In total, the body weight was 3104.05 g ± 254.07. The carcasses were then plucked and gutted. After all, the birds were slaughtered, the carcasses were transferred to a cold store (Hendi, Poznań, Poland) to be cooled at 4 °C for 24 h until meat quality analyzes were performed.

### Chemical composition of bedding material

Bedding for chemical analysis was collected in string bags on 7 dates − 1st, 7th, 14th, 21st, 28th, 35th, and 42nd rearing days. The material from the pen’s center, near its wall, and from the feeder and drinker was collected into one bag (2 bags per pen). Samples were collected from the surface to the floor (in cross-section). At one time, 200 g of material per bag was collected. Dry matter content was determined using the PN-ISO 6865:2002 (2002) method, and crude fiber content using the PN-ISO 6496:2002 (2002) method. The content of components such as nitrogen, phosphorus, and potassium was also verified using the Kjeldahl method (PN-EN ISO 6492:2005, 2005), photometric and atomic absorption spectrometry (ASA, PN-EN ISO 6869:2002, 2002), respectively. In addition, the amount of acid detergent fiber (ADF) and neutral detergent fiber (NDF) was determined following the methodological assumptions contained in PN-EN ISO 13906:2009 (2009) and PN-EN ISO 16472:2007 (2007). Bedding pH measurements were made potentiometrically.

### Growth performance and production efficiency

Birds’ body weight (BW) was measured on the first rearing day and every 7 days. Feed intake (FI) was recorded daily. Based on these results, growth rate (%) was calculated for each week of rearing, body weight gain ( $\mathrm{BWG} =final\;body\;weight \left(g\right)-initial\;body\;weight \left(g\right)$), average daily body weight gain (ADBWG = $\frac{BW \left(g\right)}{age \left(days\right)}$), average daily feed intake (ADFI = $\frac{FI \left(g\right)}{age \left(days\right)}$), feed conversion ratio (FCR = $\frac{FI \left(kg\right)}{BWG\left(kg\right) }$) for each feeding period and throughout the rearing period. In addition, the European Production Efficiency Index (EPEF = $\frac{\left(viability\;\left(\%\right)\;\times\;BW\;\left(g\right)\right)}{age\;\left(days\right)\;\times\;FCR\;\left(\frac{kg\;feed}{kg\; gain}\right)}$) and the European Performance Index (EBI = $\frac{viability\;\left(\%\right)\;\times\;ADG\;\frac{\frac{g}{chick}}{day}}{FCR \frac{kg \ feed}{kg \ gain} \times 10 }$). Calculations were made using formulas following the methods described by Biesek et al. (2022b).

### Incidence of *footpad dermatitis*

On the day of slaughter, *footpad dermatitis* in all ducks was verified based on a 5-point scale proposed by Klambeck et al. (2019). In the cited research, the photography was shown. Based on this, the examination was done. Level 0 defines the absence of lesions on the foot pad. For score 1 - slight hyperkeratosis involving < 50% of the foot pad and toes, 2 - severe hyperkeratosis/parakeratosis involving > 50% of the foot pad and toes, 3 - pododermatitis affecting > 50% of the foot pad and all toes, and 4 - severe ulcerative inflammation entire foot and toes. Scores are given as a percentage of the group with a given score.

### Carcass features and meat quality

The pH of the pectoral muscle was measured using a pH meter (Elmetron, Zabrze, Poland) with a dagger electrode 24 h after slaughter. The device was previously calibrated with standard pH 4.00, 7.00, and 9.00. The carcass and offal (heart, liver, gizzard) were weighed (Radwag, Radom, Poland), and then dissection was performed according to the method of Ziołecki and Doruchowski (1989). All selected elements were weighed, including neck, neck skin, pectoral muscles (major and minor), leg muscles (drumsticks and thighs, deboned), skin with subcutaneous fat, wings (with skin), abdominal fat, and carcass remains.

Meat color was measured using a Konica Minolta colorimeter (model CR-400) (after prior calibration), using the scale: L*- lightness, a*- redness, and b*- yellowness using the CIELab method. Drip loss was performed by placing the pectoral muscle in a string bag and suspended in a larger string bag to ensure easy water flow. Muscle weight was measured before bagging and after 24 h of storage at 4 °C. Drip loss was then calculated (Grau and Hamm 1952). Water holding capacity (WHC) was analyzed for pectoral and leg muscles minced with a meat grinder (Hendi, Poznań, Poland). A sample of 0.300 g (± 0.005 g) was weighed from each group and placed on pieces of Whatman paper (4 × 5 cm). Then the sample was covered with another piece of tissue paper and loaded with a 2 kg weight for 5 min. After this time, the sample was weighed again, and each group’s WHC of the pectoral and leg muscles was calculated (Honikel, 1987). To analyze the chemical composition (protein, intramuscular fat, salt, water), an 80 g sample of minced meat (legs and pectoral muscles) was prepared for each group. Analyzes were performed using FoodScan (FOSS) by spectrophotometry using near-infrared transmission (NIT). The protein, collagen, salt, intramuscular fat, and water content were analyzed (PN-A-82109:^2010^, ^2010^).

Texture analyses of the pectoral muscles were performed using a TA.XT plus C texture meter (Stable Micro System, Godalming, United Kingdom). Two samples measuring 1.5 × 1 × 1 cm were cut from each pectoral muscle. The meat texture was measured using firmness (N) and toughness (N×sec). For this purpose, the prepared samples were placed on a heavy-duty platform and then cut perpendicularly to the course of muscle fibers with a Warner-Bratzler flat knife. The knife speed was set to 1.5 mm/s. The second sample was previously subjected to thermal treatment at 80 °C for 40 min, cooled down, and subjected to analogous measurements as raw samples, following Gornowicz et al. (2018).

Breaking bone strength was performed using an Instron 3345 (Instron, Buckinghamshire, UK) with Bluehill 3.0 software (3345, 2013). Muscle-free tibia and femur were assessed using an appropriate attachment (Bend Future 10 mm Anvil). After the test, the result determined the maximum force necessary to break a bone (N) (Kuźniacka et al. 2017).

### Statistical calculation

The numerical data were analyzed in the statistical program Statistica 13.3. (Tibco, Statsoft, Cracow, Poland). Production results were analyzed in 5 replicates per group. The results of carcass and meat quality features were verified on 10 repetitions for each group. For *footpad dermatitis*, each duck was considered separately. The mean values of quantitative features for each group (C, CH10, CH25, CH50) and the standard error of the mean (SEM) were calculated. The normal distribution and homogeneity of the sample were verified. Dependent variables were subjected to Tukey’s post-hoc test (multiple comparisons of statistically significant differences), assuming *P* < 0.05. Tukey’s various N test was used for *footpad dermatitis*. It was related to duck viability. The bedding characteristics were analyzed depending on the rearing days and the type of bedding material. Results are presented as the interaction of bedding material (C, CH10, CH25, CH50) and rearing days (0, 7, 14, 21, 28, 35, 42), assuming *P* < 0.05. Data are also separately presented as average values for rearing days or bedding material. The adjusted coefficient of determination (adj. R^2^) for each bedding feature was also analyzed.

## Results

Table 1 presents the chemical composition of the pellet bedding with a different share of coffee husks at weekly intervals for 42 days. When comparing the data taking into account the interaction between the factors (breeding days × bedding material), statistically significant differences were found in all physicochemical characteristics of the bedding (*P* < 0.001).

**Table 1:** Physiochemical features of freshly prepared pellet bedding and during the rearing period

	Group^1^	Dry matter (%)	±SD	Crude fiber (%)	±SD	NDF^2^ (%)	±SD	ADF^3^ (%)	±SD	Nitrogen (%)	±SD	Phosphorus (%)	±SD	Potassium (%)	±SD	pH	±SD
Days or rearings × bedding	0×C	92.18^a^	0.23	47.93^a^	0.79	75.55^a^	0.64	50.53^a^	0.36	2.20^ijklm^	0.09	0.06^ L^	0.01	0.34^ m^	0.03	6.51^bcde^	0.05
	0×CH10	90.98^a^	0.30	44.97^b^	1.25	76.22^a^	0.71	50.32^a^	1.52	3.20^b^	0.12	0.05^ L^	0.00	0.39^ m^	0.03	6.57^bc^	0.06
	0×CH25	91.13^a^	1.13	42.84^bc^	0.77	71.01^b^	1.60	47.56^ab^	1.04	3.51^a^	0.28	0.06^ L^	0.00	0.64^kl^	0.03	6.76^a^	0.05
	0×CH50	90.78^a^	0.21	37.40^d^	0.50	62.51^e^	1.15	42.36^ cd^	0.59	3.50^a^	0.16	0.06^ L^	0.00	0.85^ij^	0.10	6.73^a^	0.05
	7×C	73.76^bc^	1.28	42.13^bc^	2.40	69.12^bc^	2.44	44.45^bc^	1.75	2.53^cdefgh^	0.37	0.24^k^	0.06	0.55^ L^	0.08	5.80^hi^	0.12
	7×CH10	71.33^c^	1.27	40.30^ cd^	1.96	67.87^ cd^	2.55	42.82^ cd^	2.17	2.51^defgh^	0.34	0.22^k^	0.08	0.57^kl^	0.06	5.68^i^	0.34
	7×CH25	75.45^bc^	2.78	40.68^c^	0.69	66.95^d^	1.61	43.96^c^	1.63	2.19^ijklm^	0.21	0.17^k^	0.03	0.65^k^	0.05	5.39^j^	0.10
	7×CH50	79.07^b^	1.43	33.75^e^	1.29	57.08^fgh^	1.51	40.09^de^	0.74	2.62^cdef^	0.22	0.20^k^	0.05	0.91^ij^	0.05	5.36^j^	0.09
	14×C	53.65^ef^	5.45	32.89^efg^	1.65	63.55^e^	1.64	37.95^ef^	2.43	1.96^ m^	0.10	0.57^hij^	0.05	0.93^i^	0.02	6.40^def^	0.02
	14×CH10	58.08^de^	4.89	32.50^efg^	2.42	57.60^ fg^	0.70	36.91^efg^	1.74	2.00^ lm^	0.15	0.51^ij^	0.03	0.82^j^	0.07	6.38^def^	0.13
	14×CH25	75.94^bc^	5.87	31.21^efgh^	5.38	56.50^fghi^	0.64	35.25^fghij^	0.85	2.01^ lm^	0.09	0.50^j^	0.04	0.88^ij^	0.05	6.58^bc^	0.04
	14×CH50	60.86^d^	2.44	29.90^hij^	1.91	54.20^ijk^	1.94	35.47^fghij^	0.40	2.30^ghijkl^	0.08	0.55^hij^	0.08	1.14^ h^	0.05	6.63^ab^	0.05
	21×C	48.69^f^	4.33	29.71^hij^	0.72	54.45^ij^	0.99	30.98^klm^	3.25	2.49^defghi^	0.10	0.94^e^	0.05	1.17^ h^	0.07	5.88^ h^	0.05
	21×CH10	71.18^c^	8.21	28.70^hijkl^	0.53	58.38^f^	0.38	35.48^fghij^	2.04	2.33^fghijk^	0.22	0.59^hi^	0.04	0.86^ij^	0.04	5.86^ h^	0.02
	21×CH25	74.25^bc^	2.80	28.16^ijkl^	0.75	57.60^ fg^	0.84	32.21^jklm^	3.90	2.55^cdefg^	0.16	0.60^gh^	0.03	0.93^i^	0.01	5.75^hi^	0.06
	21×CH50	63.79^d^	5.06	28.53^hijkl^	0.38	50.26^lmno^	0.93	26.84^n^	0.73	2.58^cdef^	0.06	0.67^ g^	0.08	1.11^ h^	0.01	5.81^hi^	0.01
	28×C	22.04^ g^	1.39	26.47^klm^	1.41	52.20^ L^	0.75	36.22^fgh^	2.56	2.63^cdef^	0.19	1.14^ab^	0.06	1.54^de^	0.07	6.22^ g^	0.06
	28×CH10	24.97^ g^	3.15	27.01^jklm^	0.95	52.23^kl^	0.87	35.37^fghij^	3.43	2.55^cdefg^	0.16	1.11^bc^	0.07	1.44^ fg^	0.04	6.29^ fg^	0.08
	28×CH25	27.35^ g^	6.35	25.94^ lm^	1.05	49.94^mno^	0.66	35.36^fghij^	2.84	2.78^ cd^	0.13	1.10^bc^	0.08	1.43^ g^	0.05	6.41^def^	0.08
	28×CH50	24.17^ g^	3.31	24.83^ m^	0.81	48.70^no^	0.95	34.43^ghij^	2.34	2.83^c^	0.21	1.20^a^	0.04	1.58^cde^	0.04	6.45^cdef^	0.07
	35×C	23.57^ g^	1.97	30.38^ghi^	1.56	54.59^ij^	1.13	35.95^fghi^	2.04	2.17^jklm^	0.15	1.05^ cd^	0.04	1.56^de^	0.08	6.39^def^	0.07
	35×CH10	23.18^ g^	0.88	28.44^hijkl^	3.37	55.51^hij^	0.62	34.12^ghijk^	1.89	2.41^fghij^	0.06	1.15^ab^	0.04	1.53^def^	0.04	6.37^efg^	0.11
	35×CH25	26.03^ g^	3.28	28.01^ijkl^	0.90	54.49^ij^	0.93	32.34^jklm^	2.14	2.52^defg^	0.08	1.10^bc^	0.05	1.52^efg^	0.05	6.44^cdef^	0.10
	35×CH50	24.05^ g^	1.74	27.36^jklm^	0.49	50.84^ lm^	0.64	32.62^ijkl^	0.75	2.63^cdef^	0.10	1.11^bc^	0.03	1.69^b^	0.06	6.41^def^	0.06
	42×C	27.39^ g^	2.05	32.31^efg^	1.88	53.59^jkl^	0.91	33.30^hijkl^	2.56	2.09^klm^	0.11	0.80^f^	0.03	1.53^def^	0.11	6.53^bcd^	0.05
	42×CH10	26.38^ g^	4.85	30.56^ghi^	1.04	55.80^ghi^	0.61	30.18^lmn^	0.72	2.25^ghijklm^	0.23	1.00^de^	0.04	1.68^bc^	0.09	6.53^bcd^	0.05
	42×CH25	26.73^ g^	3.10	29.04^hijk^	2.61	48.41^o^	0.47	28.97^mn^	1.81	2.35^fghijk^	0.28	1.01^de^	0.08	1.62^bcd^	0.05	6.63^ab^	0.04
	42×CH50	26.48^ g^	3.53	29.32^hijk^	0.57	50.43^lmn^	0.80	30.25^lmn^	3.18	2.47^fghij^	0.09	1.01^de^	0.02	1.81^a^	0.02	6.64^ab^	0.06
	P-value	< 0.001		< 0.001		< 0.001		< 0.001		< 0.001		< 0.001		< 0.001		< 0.001	
	Adj. R^2^	0.981		0.923		0.978		0.897		0.817		0.985		0.983		0.945	
	SEM	1.56		0.38		0.48		0.39		0.03		0.02		0.03		0.02	
Days of rearing’	0	91.27^a^	0.80	43.28^a^	3.98	71.32^a^	5.64	47.69^a^	3.47	3.10^a^	0.57	0.06^f^	0.01	0.55^e^	0.21	6.64^a^	0.12
	7	74.90^b^	3.34	39.21^b^	3.66	65.25^b^	5.24	42.83^b^	2.33	2.46^c^	0.33	0.20^e^	0.06	0.67^d^	0.16	5.56^e^	0.27
	14	62.13^c^	9.67	31.62^c^	3.30	57.96^c^	3.73	36.39^c^	1.87	2.07^d^	0.17	0.53^d^	0.06	0.94^c^	0.13	6.50^b^	0.13
	21	64.48^c^	11.30	28.77^d^	0.83	55.17^d^	3.33	31.38^e^	4.10	2.49^c^	0.17	0.70^c^	0.15	1.02^c^	0.13	5.82^d^	0.06
	28	24.63^d^	4.27	26.06^e^	1.32	50.77^f^	1.72	35.35^ cd^	2.79	2.70^b^	0.20	1.14^a^	0.07	1.50^b^	0.08	6.34^c^	0.12
	35	24.21^d^	2.34	28.55^d^	2.17	53.86^de^	1.99	33.76^d^	2.25	2.43^c^	0.20	1.10^a^	0.05	1.57^ab^	0.09	6.40^c^	0.09
	42	26.74^d^	3.42	30.31^ cd^	2.10	52.06^ef^	2.96	30.67^e^	2.71	2.29^c^	0.23	0.95^b^	0.10	1.66^a^	0.13	6.58^ab^	0.07
	P-value	< 0.001		< 0.001		< 0.001		< 0.001		< 0.001		< 0.001		< 0.001		< 0.001	
	Adj. R^2^	0.944		0.821		0.774		0.801		0.502		0.958		0.898		0.885	
Bedding	C	48.76	25.23	34.54^a^	7.28	60.44^a^	8.60	38.48^a^	6.69	2.30^c^	0.29	0.68	0.39	1.09^b^	0.47	6.25	0.29
	CH10	52.30	25.90	33.21^a^	6.59	60.51^a^	7.97	37.89^a^	6.51	2.46^bc^	0.39	0.66	0.41	1.04^b^	0.47	6.24	0.35
	CH25	56.69	26.96	32.27^ab^	6.64	57.84^a^	7.87	36.52^ab^	6.65	2.56^ab^	0.49	0.65	0.41	1.09^b^	0.39	6.28	0.48
	CH50	52.74	26.13	30.16^b^	4.02	53.43^b^	4.72	34.58^b^	5.25	2.70^a^	0.39	0.68	0.42	1.30^a^	0.37	6.29	0.48
	P-value	0.355		< 0.001		< 0.001		0.002		< 0.001		0.937		0.002		0.867	
	Adj. R^2^	0.009		0.052		0.122		0.044		0.114		0.002		0.041		0.002	

^a,b^, different letters in the row show statistically significant differences between the groups, P-value < 0.05; ^1^, C, the control group kept on the wheat straw bedding; CH10, the group kept on the bedding with 10% of coffee husks and 90% of wheat straw; CH25, the group kept on the bedding with 25% of coffee husks and 75% of wheat straw; CH50, the group kept on the bedding with 50% of coffee husks and 50% of wheat straw; Adj. R^2^, adjusted determination coefficient; SEM; standard error of the mean; ^2^, NDF, neutral detergent fiber; ^3^, ADF, acid detergent fiber

Dry matter in all groups (C - CH50) was significantly highest in fresh bedding (day 0) compared to the other rearing days (in each group). Significantly the lowest dry matter was found in all types of bedding from day 28 to the end of rearing, compared to pellets on days 0 to 21 (*P* < 0.001). The highest crude fiber content was recorded in fresh straw pellets (group C) than in other groups. The lowest content was in the CH50 group on the 28th day of rearing (*P* < 0.001). When analyzing the content of NDF, the significantly highest value in groups C and CH10 in fresh material and the lowest in group CH25 on day 42 was noticed (*P* < 0.001). The highest ADF content was found in fresh bedding from groups C and CH10 compared to other values, except for the CH25 group from day 0. The lowest ADF content was found in the CH50 group from the 21st day of rearing, compared to other groups, except groups CH10, CH25, and CH50 on day 42 (*P* < 0.001).

In the CH25 and CH50 groups (day 0), nitrogen content was significantly higher than the CH10 group (day 0). The indicated groups had a significantly higher nitrogen content than the others. The lowest value was shown in group C on the 14th rearing day (*P* < 0.001). In all groups (C - CH50), fresh bedding (day 0) showed significantly the lowest phosphorus content compared to other groups at all dates (*P* < 0.001). Then, phosphorus content was increased on the 28th day, with its significantly highest content in group C and CH50 (*P* < 0.001). In the following days, the value decreased again. On the other hand, fresh bedding from groups C and CH10 was characterized by significantly the lowest potassium content (*P* < 0.001) compared to all groups at all dates. The highest potassium content was found in the CH50 group on the 42nd day of chicken rearing (*P* < 0.001). There was an increasing trend in potassium content during rearing, significantly from day 7 to the end of rearing, and a significant difference can be seen between all groups (C - CH50) up to day 21 and from day 28 (*P* < 0.001). The highest pH value was found in fresh bedding from the CH25 and CH50 groups, compared to the other groups (*P* < 0.001), except for CH50 on day 14 and CH25 and CH50 on day 42 (P > 0.05). The lowest pH values were found on day 7 in the CH25 and CH50 groups (*P* < 0.001). When comparing all groups on all dates, a trend of differences in the pH of the bedding was noted, with an increase in pH value on the 28th day after its previous decrease on the 21st day (*P* < 0.001). High adj. R^2^ has been demonstrated for all features ranging from 0.817 to 0.985.

When analyzing the chemical composition, according to rearing days, significant differences for each feature were found (*P* < 0.001). Higher dry matter content was shown in fresh bedding. A decreasing trend was demonstrated in the following days; from day 28, it remained at a similar level (< 25%). The highest crude fiber content was found in fresh bedding, as was the NDF and ADF content (*P* < 0.001). Significantly the lowest content of crude fiber and NDF was found on day 28, ADF on days 21 and 42 (*P* < 0.001). Nitrogen content was higher in fresh material, significantly decreased until day 14, and increased on days 21 and 28 (*P* < 0.001). On the 35th and 42nd days, it significantly decreased compared to the 28th day of rearing (*P* < 0.001). The lowest content of phosphorus was found in the fresh material. An upward trend to 28–35 days was noticed and decreased again on day 42 (*P* < 0.001). Similarly, changes in the content of potassium were found. The highest content characterized the material on the 42nd day of rearing (*P* < 0.001). The pH value, relative to days, was highest in fresh material (day 0) and lowest on day 7 (*P* < 0.001). High and very high adj. R^2^ has been demonstrated for features tested according to rearing days (from 0.502 to 0.944).

When comparing the average values with the bedding material, statistically significant differences were found in the content of crude fiber, NDF, ADF, nitrogen, and potassium (*P* < 0.05). The crude fiber and ADF content were significantly higher in group C and CH10 than in CH50 (*P* < 0.001, P = 0.002, respectively). The CH50 group showed significantly lower NDF content than the other groups (*P* < 0.001). Nitrogen content was higher in the CH50 group than in C and CH10 and in CH25 compared to C (*P* < 0.001). The potassium content in the CH50 group was significantly higher than in the other groups (P = 0.002). When analyzing the characteristics concerning the bedding material, a low adj. R^2^ was shown (0.002 to 0.122).

The production results of ducks are presented in Table 2. The highest growth rate (%) was found for ducks from the CH25 group (P = 0.009) compared to the other groups in the last week of rearing. For other features, no statistically significant differences between the groups were found (P > 0.05). *Footpad dermatitis* (FPD) in ducks was also verified on the day of slaughter (Table 3). There were no statistically significant differences between the groups (P > 0.05).

**Table 2:** Growth performance of broiler ducks

Item^1^		Group^2^				SEM^3^	P-value
		C	CH10	CH25	CH50		
Viability (%)		90.00	88.00	94.00	94.00	2.21	0.742
BW (g)	day 1	54.72	56.66	56.36	56.52	0.33	0.117
	Day 7	235.91	242.77	234.57	236.04	2.13	0.554
	Day 14	715.91	732.46	698.09	721.51	5.45	0.153
	Day 21	1352.29	1426.94	1365.16	1433.91	14.00	0.067
	Day 28	2073.91	2155.22	2007.62	2068.26	21.04	0.088
	Day 35	2553.01	2680.94	2558.33	2521.24	41.56	0.577
	Day 42	2968.11	3110.64	3179.97	2931.61	52.22	0.301
The growth rate (%)	Week 1	124.63	124.24	122.42	122.72	0.59	0.492
	Week 2	100.89	100.43	99.39	101.40	0.45	0.470
	Week 3	61.39	64.32	64.67	66.08	0.82	0.232
	Week 4	42.16	40.60	38.12	36.18	0.95	0.109
	Week 5	20.50	21.67	23.77	19.69	1.23	0.705
	Week 6	14.98^b^	14.87^b^	21.73^a^	14.96^b^	0.95	0.009
BWG (g)	Days 1–28	2019.19	2098.56	1951.26	2011.74	21.01	0.089
	Days 29–42	894.20	955.41	1172.35	863.35	49.78	0.107
	Total	2913.39	3053.98	3123.61	2875.09	52.18	0.303
ADBWG (g)	Days 1–28	72.11	74.95	69.69	71.85	0.75	0.089
	Days 29–42	68.78	73.49	90.18	66.41	3.83	0.107
	Total	69.37	72.71	74.37	68.45	1.24	0.303
FI (g)	Days 1–28	3939.28	4107.10	3939.19	4018.86	42.01	0.467
	Days 29–42	4151.36	4221.11	4050.52	4059.78	77.95	0.869
	Total	8171.52	8328.21	8075.13	8078.64	112.46	0.864
ADFI (g)	Days 1–28	253.76	293.36	281.37	287.06	7.98	0.327
	Days 29–42	219.81	201.01	192.88	193.32	5.90	0.352
	Total	194.56	198.29	192.27	192.35	2.68	0.864
FCR (kg/kg)	Days 1–28	1.95	1.96	2.02	2.00	0.02	0.462
	Days 29–42	4.84	4.50	3.59	4.84	0.23	0.175
	Total	2.82	2.73	2.60	2.82	0.05	0.321
EPEF		227.78	240.21	277.01	233.80	9.84	0.300
EBI		223.56	235.84	272.12	229.29	9.70	0.297

^a,b^, different letters in the row show statistically significant differences between the groups, P-value < 0.05; ^1^, BW, body weight; BWG, body weight gain; ADBWG, average daily body weight gain; FI, feed intake; ADFI, average daily feed intake; FCR, feed conversion ratio; EPEF, European Production Efficiency Factor; EBI, European Broiler Index; ^2^, C, the control group kept on the wheat straw bedding; CH10, the group kept on the bedding with 10% of coffee husks and 90% of wheat straw; CH25, the group kept on the bedding with 25% of coffee husks and 75% of wheat straw; CH50, the group kept on the bedding with 50% of coffee husks and 50% of wheat straw; ^3^, standard error of the mean

**Table 3:** Incidence of *footpad dermatitis* score in duck flocks (%)

Item^1^	Group^2^				SEM^3^	P-value
	C	CH10	CH25	CH50		
FPD score 0	0.00	5.00	2.50	2.50	1.46	0.725
FPD score 1	30.29	38.70	36.28	12.50	7.25	0.608
FPD score 2	33.27	28.94	32.94	22.94	3.95	0.803
FPD score 3	21.56	10.00	19.61	27.39	3.65	0.428
FPD score 4	14.89	17.36	8.67	34.67	4.41	0.189

No statistically significant differences between the groups were noticed, P-value > 0.05; each duck was assessed individually within the pens; the results are presented as a percentage of the group. ^1^, FPD score: 0, no alternations; 1, slight hyperkeratosis on either < 50% of the footpad or toepads; 2, severe hyperkeratosis/parakeratosis on either > 50% of footpad or > 50% of the toepads; 3, superficial pododermatitis on > 50% of the footpad and the whole toepads; 4, severe ulcerative pododermatitis on the whole footpads and toepads; ^2^, C, the control group kept on the wheat straw bedding; CH10, the group kept on the bedding with 10% of coffee husks and 90% of wheat straw; CH25, the group kept on the bedding with 25% of coffee husks and 75% of wheat straw; CH50, the group kept on the bedding with 50% of coffee husks and 50% of wheat straw; ^3^, standard error of the mean

The carcass characteristics of ducks are presented in Table 4. Birds in the CH25 group had the highest pre-slaughter weight compared to groups C and CH50 (P = 0.002). Significantly higher carcass weight and carcass weight with offal were found in the CH10 and CH25 groups compared to group C. In the CH25 group, significantly higher liver weight was also found (P = 0.043), and a lower percentage of the neck in the carcass (P = 0.020). Significantly lower pectoral muscle weight was found in group C compared to CH10 (P = 0.036), in contrast to the percentage of wings in the carcass (P = 0.012). Lower carcass’ remains weight was found in groups C and CH50 (P = 0.006).

**Table 4:** Carcass features of broiler ducks

Item	Group^1^				SEM^2^	P-value
	C	CH10	CH25	CH50		
Pre-slaughter body weight (g)	2907.50^c^	3188.50^ab^	3289.70^a^	3030.50^bc^	40.17	0.002
Carcass weight (g)	1906.85^b^	2148.91^a^	2202.08^a^	1990.71^ab^	34.07	0.004
Slaughter yield (%)	65.43	67.44	66.88	65.69	0.46	0.356
Carcass with offal weight (g)	2064.92^b^	2313.98^a^	2379.97^a^	2155.96^ab^	34.86	0.002
Slaughter yield with offal (%)	70.90	72.62	72.30	71.14	0.42	0.387
Heart (g)	17.14	19.16	17.77	16.52	0.47	0.225
Heart (%)	0.91	0.89	0.80	0.83	0.02	0.227
Liver (g)	62.02^b^	66.36^ab^	77.35^a^	68.53^ab^	2.00	0.043
Liver (%)	3.34	3.10	3.53	3.44	0.11	0.522
Gizzard (g)	78.91	79.55	82.77	80.20	1.37	0.782
Gizzard (%)	4.17	3.71	3.78	4.05	0.08	0.154
Neck (g)	154.57	162.00	159.66	165.46	3.03	0.652
Neck (%)	8.16^ab^	7.54^ab^	7.25^b^	8.32^a^	0.14	0.020
Pectoral muscle (g)	340.55^b^	422.72^a^	410.66^ab^	368.94^ab^	11.51	0.036
Pectoral muscle (%)	17.62	19.63	18.64	18.53	0.38	0.337
Leg muscle (g)	272.38	274.34	287.35	276.68	6.72	0.873
Leg muscle (%)	14.32	12.74	13.04	13.93	0.28	0.155
Total muscle (g)	612.93	697.06	698.01	645.62	14.66	0.105
Total muscle (%)	31.94	32.37	31.68	32.46	0.37	0.873
Skin with subcutaneous fat (g)	370.98	443.50	450.40	392.62	12.60	0.062
Skin with subcutaneous fat (%)	19.41	20.63	20.41	19.51	0.41	0.651
Abdominal fat (g)	13.05	17.63	15.46	13.74	1.22	0.568
Abdominal fat (%)	0.67	0.81	0.71	0.68	0.05	0.766
Fatness (g)	384.03	461.13	465.86	406.36	13.30	0.067
Fatness (%)	20.08	21.44	21.11	20.18	0.43	0.627
Wings with skin (g)	249.05	244.95	270.07	248.57	4.10	0.117
Wings with skin (%)	13.10^a^	11.42^b^	12.32^ab^	12.51^ab^	0.19	0.012
Carcass remains (g)	506.27^b^	583.77^ab^	608.48^a^	524.70^b^	12.39	0.006
Carcass remains (%)	26.72	27.23	27.63	26.53	0.50	0.875

^a,b^, different letters in the row show statistically significant differences between the groups, P-value < 0.05; ^1^, C, the control group kept on the wheat straw bedding; CH10, the group kept on the bedding with 10% of coffee husks and 90% of wheat straw; CH25, the group kept on the bedding with 25% of coffee husks and 75% of wheat straw; CH50, the group kept on the bedding with 50% of coffee husks and 50% of wheat straw; ^2^, standard error of the mean

The physicochemical properties of the pectoral and leg muscles and leg bone strength are presented in Table 5. The CH25 group pectoral muscles were significantly characterized by the highest water retention capacity (*P* < 0.001) and the percentage of water content (*P* < 0.001). The highest protein content was found in group C compared to the other groups (*P* < 0.001). Significantly the lowest content of collagen, salt, and intramuscular fat was found in the pectoral muscles of ducks from the CH25 group (*P* < 0.001). The highest water-holding capacity was demonstrated in the leg muscles of ducks from the CH25 and CH50 groups (P = 0.004), which at the same time did not differ significantly from the muscles from the CH10 group. The highest protein and water content characterized leg muscles from group C compared to the experimental groups (*P* < 0.001). The percentage of salt was significantly highest in leg muscles in the CH10 group and intramuscular fat in the CH50 group (*P* < 0.001).

**Table 5:** Physicochemical features of broiler ducks’ muscles and the breaking strength of the leg bones

Item^1^		Group^2^				SEM^3^	P-value
		C	CH10	CH25	CH50		
Pectoral muscle	pH_24_	6.15	6.17	6.17	6.14	0.02	0.884
	L* - color	40.40	38.73	38.65	38.66	0.45	0.455
	a* - color	14.43	15.27	16.04	15.94	0.25	0.080
	b* - color	2.48	2.62	2.70	2.65	0.25	0.992
	Drip loss (%)	1.40	1.29	1.97	1.90	0.13	0.139
	Water-holding capacity (%)	34.98^ab^	29.62^bc^	39.14^a^	28.85^c^	0.97	< 0.001
	Protein (%)	21.14^a^	21.04^b^	21.03^b^	20.85^c^	0.02	< 0.001
	Collagen (%)	1.59^a^	1.63^a^	1.18^b^	1.55^a^	0.03	< 0.001
	Salt (%)	0.38^a^	0.35^ab^	0.23^c^	0.31^b^	0.01	< 0.001
	Intramuscular fat (%)	2.87^b^	3.00^a^	2.05^c^	3.03^a^	0.06	< 0.001
	Water (%)	77.21^b^	76.47^c^	77.53^a^	76.23^d^	0.09	< 0.001
	Firmness of raw muscle (N)	75.12	69.21	53.79	55.20	4.14	0.190
	Toughness of raw muscle (N×sec)	454.05	447.40	344.38	363.56	24.73	0.286
	Firmness of cooked muscle (N)	46.43	43.27	52.88	52.92	2.15	0.292
	Toughness of cooked muscle (N×sec)	331.87	272.18	321.79	328.10	14.22	0.424
Leg muscle	L* - color	37.08	36.33	37.32	36.54	0.49	0.888
	a* - color	13.97	13.81	15.30	15.35	0.34	0.216
	b* - color	1.98	2.26	2.96	2.90	0.20	0.246
	Water-holding capacity (%)	30.71^b^	32.61^ab^	34.25^a^	34.29^a^	0.42	0.004
	Protein (%)	19.48^a^	18.63^d^	18.79^c^	19.05^b^	0.05	< 0.001
	Collagen (%)	1.72	1.55	1.68	1.73	0.02	0.356
	Salt (%)	0.60^b^	0.69^a^	0.53^c^	0.59^b^	0.01	< 0.001
	Intramuscular fat (%)	5.31^d^	6.53^b^	6.08^c^	6.83^a^	0.09	< 0.001
	Water (%)	74.81^a^	74.03^c^	74.14^b^	72.95^d^	0.11	< 0.001
Breaking strength of tibia bone (N)		254.74	235.62	288.11	270.49	9.33	0.232
Breaking strength of femur bone (N)		270.88	239.15	228.67	246.12	7.60	0.247

^a,b^, different letters in the row show statistically significant differences between the groups, P-value < 0.05; ^1^, L* - lightness; a* - redness; b* - yellowness; ^2^, C, the control group kept on the wheat straw bedding; CH10, the group kept on the bedding with 10% of coffee husks and 90% of wheat straw; CH25, the group kept on the bedding with 25% of coffee husks and 75% of wheat straw; CH50, the group kept on the bedding with 50% of coffee husks and 50% of wheat straw; ^3^, standard error of the mean

## Discussion

Conducting a physicochemical analysis of the bedding after the production cycle is essential in assessing its future suitability for use as a natural fertilizer in plant production (Rogeri et al. 2016). Our research found higher nitrogen and potassium content in the CH50 group and phosphorus in all experimental groups compared to group C on the 42nd day. Poultry manure may vary due to the type of feed used, feed additives, bedding, and storage (Bolan et al. 2010). Bedding after rearing chickens, a mixture of wood dust or rice hulls, feed, and feces, was characterized by a lower dry matter (66%) and higher nitrogen, potassium, and phosphorus content (Rogeri et al. 2016). It depends on the type of bedding used and the ability of the birds to use the feed. It is assumed that the nitrogen and phosphorus content is higher in chicken manure than in fresh bedding (Bolan et al. 2010), whereas, in our research, such an effect was in phosphorus and potassium content. According to Kılıc and Guleciuz (2017), wheat straw contains 76.68% NDF and 46.89% ADF.

On the other hand, according to Badarina et al. (2013), coffee husks contained 95.17% and 87.18% of ADF and NDF in dry matter, respectively. In the CH50 group, the value of NDF and ADF was significantly the lowest compared to the other groups. In the litter after 6 weeks of chicken rearing, the NDF was 54.2%, and the ADF was 27.3% (Bakshi and Fontenot 1998). This trend is consistent with our results and may be due to the high proportion of duck feces in the samples for testing on day 42. In our research, on day 21, pH decreased. However, since day 28, was increasing. A lower pH of the litter may improve its quality due to the reduction of enzyme activity and an unfavorable environment for the development of bacteria that convert nitrogen compounds into ammonia (NH_3_), which is one of the factors affecting the welfare of birds (Toppel et al. 2019).

As pointed out by Brink et al. (2022), high clumping of bedding may explain the significantly lower percentage of nitrogen and uric acid due to reduced water evaporation and oxygen availability, which affected the limited conversion of nitrogen compounds to ammonia. In our research, the pellets (in each group) at the end of rearing contained high moisture, which may have contributed to the high level of caking. Nitrogen content was also lower in bedding than at the beginning of rearing. The increased moisture also affected the increasing pH since the 4th week of rearing.

The highest growth rate of ducks was shown in the CH25 group in the last week of rearing. The influence of the type of litter on the relative growth rate was also found in the studies of Mohammed et al. (2019). As the authors indicate, this may be related to the more intense manifestation of specific bird behaviors, particularly those related to feed intake. Eser et al. (2022) compared wood shavings and paper waste sludge (PWS). As indicated by the authors, the bedding was a mixture of PWS and sepiolite (50:50) to reduce the risk of too high humidity when using only PWS. The birds’ highest body weight and weight gain (*P* < 0.001) was observed on the 42nd rearing day in the group where this type of bedding and wood shavings were used. There was also no negative effect of PWS and sepiolite on selected carcass features and physicochemical parameters of pectoral and leg muscles. Similar conclusions were reported by Onbaşılar (2022). The study also used PWS and sepiolite as bedding materials in broiler chickens. The author stated that bedding affects birds’ well-being and natural behavior. Using alternative waste materials has been shown to increase the percentage of walking and resting behaviors.

The severity of FPD changes indicates flock health and birds’ well-being. So far, the type of bedding material used has been of great importance in the occurrence of this lesion. Findings of intense changes in FPD in broiler chickens kept on coconut husk or grass at 10.2% and 11.4%, respectively. In contrast, no such changes were noted in wood chips or rice husks (Garcês et al. 2017). Compared to plastic flooring, a lower percentage of FPD in ducks was found with wood chip bedding. Changes at levels 2, 3, and 4 were shown (De Almeida et al. 2017). According to Oke et al. (2019), the type of bedding did not affect the occurrence of FPD in broiler chickens using wood chips, cut corn cobs, and Panicum maximum. According to Qin et al. (2019), FPD in ducks may also increase the inflammatory response of birds (higher concentrations of endotoxins in plasma and interleukins) and affect changes in the cecal microbiome, which is of great importance in terms of herd health.

Barbosa et al. (2022) studied the influence of different litter compositions on broiler chicken locomotion. The authors used wood shavings, rice husks, and grass with different lawn grass (*Zoysia japonica*) ratios. The pododermatitis on various days was evaluated. The lower presence of FPD was in the group with rice husks and different levels of grass. However, in our research, even severe ulcerative pododermatitis on the whole footpads and toepads in each group. Our observations indicated that this situation might result from a high moisture content (since the 28th day) of the bedding due to splashing a large amount of water from the drinkers during its intake by the birds. Barbosa et al. (2022) discussed that pododermatitis’s severity increases with bedding moisture increasing. The moisture softens and opens the footpad skin. It results in immune system reaction and dermatitis causing. The negative impact on pododermatitis was from the 28th day. It corresponds with our results. In contrast, Boussaada et al. (2022) concluded that inflammation of the foot pads and ankle joints was not correlated with the physicochemical parameters of the bedding (pH, NH_3_, moisture).

When analyzing the characteristics of duck carcasses, the highest pre-slaughter weight, carcass weight with offal, and liver weight were found in the CH25 group. Nevertheless, the slaughter yield was similar in all groups, which indicates that the bedding had no adverse effect on the carcass. The other types of bedding impact were analyzed (wood chips, sand, coconut husk, rice husks, grass, corncobs, and newspapers), and the authors found a minor effect on the final body weight of birds (Garcês et al. 2017). As cited authors noted, possible changes in carcass characteristics of chickens were attributed to the association with the incidence of *footpad dermatitis*, which limited the ability to move freely and eat feed.

According to Oke et al. (2019), the bedding did not affect meat’s protein and fat content, unlike our research, where significant differences between the groups were found in all analyzed chemical components (except for collagen in the leg muscles). The most favorable results for water-holding capacity (WHC) were found in groups CH50 (pectoral muscles) and C (leg muscles). WHC is one of the main features influencing consumer preferences when choosing a product and testing its freshness. Significant importance for changes in WHC is also attributed to glycolysis, denaturation of muscle proteins, and shortening of sarcomeres during *rigor mortis* (Bowker 2017). However, as cited author indicated, WHC is related to many factors. In particular, attention is paid to the environment in the livestock building (e.g., humidity, temperature). It contributes to the potential occurrence of stress (thermal or oxidative). Oxidative stress reduces the activity of calcium ion transfer proteins, disturbs muscle homeostasis, and thus reduces WHC. FPD, which occurred in all groups at different levels, can also be a stress factor (Bowker 2017). It could affect the different WHC values in the pectoral and leg muscles. Significantly higher intramuscular fat content (IMF) was found in the CH50 group for pectoral muscles and leg muscles, which may be a good feature since IMF largely determines the palatability of poultry meat (Zhao et al. 2007). So far, changes in IMF content in ducks depending on the housing system have been confirmed (Guo et al. 2020). With age, increased content of IMF was found in the pectoral muscles of birds kept on the net and in the leg muscles of ducks from the bedding system (sawdust bedding). In addition, the authors also pointed to a higher content of monounsaturated and polyunsaturated fatty acids in the muscles of ducks kept on the grate.

Environmental factors can also influence the deposition of fat in poultry meat. Too high a temperature can lead to heat stress. The studies conducted in this area are inconsistent, as they indicate both an increase and a decrease in the fat content in the meat of broiler chickens (Tůmová and Teimouri 2010). The stress factor in our research may be the bedding moisture and the occurrence of FPD in all groups at different levels, resulting in variability of the IMF content in the pectoral and leg muscles. It can also be assumed that carbohydrate metabolism has been disturbed, which is the most responsible for IMF formation in the muscles (Luo et al. 2022).

Broilers spend their entire lives on bedding. The bedding management strategy is crucial (Onbaşılar 2022). According to the cited author, the behavior of birds is part of the interaction with the environment. Therefore, the behavior of birds and their well-being will affect the efficiency of production and the quality of the raw material. Toghyani et al. (2010) found that different bedding materials affected feed and water intake in birds. Feed, water intake, and bedding quality can influence stress levels in birds (Yerpes et al. 2020). Our study’s results regarding meat quality and carcass traits could have resulted from individual ducks’ perceptions of stress stimuli, e.g., bedding moisture. Stress, related to the environmental conditions of birds (e.g., stocking density), affects carcass characteristics and meat quality (Nasr et al. 2021). A study on duck housing systems showed that it is essential for productivity, carcass characteristics, and meat quality, which is related to the immune and antioxidant status (Ghanima et al. 2020).

No economic analysis was performed in the presented study. The bedding material (straw and coffee husks) was obtained free of charge. The only costs that could affect the profitability of production concerned electricity and work done in producing pellets.

In conclusion, after 42 days of broiler duck rearing, a significantly higher nitrogen and potassium content was found in the manure after the duck production cycle in the CH50 group compared to the other groups. The phosphorus content was also significantly higher in the experimental groups than in the control group, which may be necessary for further management. A significant decrease in bedding dry matter after 28 days was unbeneficial, possibly related to the ducks’ behavior (water intake). Further actions should be taken to improve this indicator. Considering the characteristics of the carcass, it is worth paying attention to the higher carcass weight (CH10 and CH25) and pectoral muscle weight (CH10) in selected groups where coffee husks in the bedding were used. However, considering the slaughter yield, it should be concluded that the different bedding materials had no adverse effect. The pectoral muscles from the CH50 group showed a significantly better ability to hold water and the highest content of IMF (similar to the leg muscles), which may determine the technological suitability of the meat and its sensory properties. No negative impact on production efficiency results allows for the reuse of waste - coffee husks, as support for pro-environmental activities. Using the regression tool (adj. R^2^) made it possible to conclude that the primary determinant was the time (rearing period) for the characteristics of the bedding material. These changes are also related to feed residues and, above all, duck manure. Thus, the coffee husks could be used in any variant (10, 25, 50%), depending on availability. Nevertheless, due to the foot pads’ condition in all groups, further research should be continued, which could be related to the dry matter of bedding on days 28–42.

## Data Availability

The datasets generated and analyzed during the current study are not publicly available due to all the results in the form of means and statistics presented in this paper. Still, it is available from the corresponding author at a reasonable request.
